# Exogenous leptin affects sperm parameters and impairs blood testis barrier integrity in adult male mice

**DOI:** 10.1186/s12958-018-0368-4

**Published:** 2018-05-31

**Authors:** Xiaotong Wang, Xiaoke Zhang, Lian Hu, Honggang Li

**Affiliations:** 1 Family Planning Research Institute/Center of Reproductive Medicine, Tongji Medical College, Huazhong University of Science and Technology, Wuhan, 430030 China; 2 Center for Reproductive Medicine, The Third Affiliated Hospital of Zhengzhou University, Zhengzhou, 450052 China

**Keywords:** Obesity, Leptin, Male infertility, Mice, Sertoli cell, Blood testis barrier

## Abstract

**Background:**

Serum leptin levels are augmented in obese infertile men and in men with azoospermia. They also correlate inversely with sperm concentration, motility and normal forms. The mechanisms underlying the adverse effects of excess leptin on male reproductive function remain unclear. The present study aimed to evaluate the effects of exogenous leptin on sperm parameters in mice and to explore the underlying mechanisms.

**Methods:**

We treated normal adult male mice with saline, 0.1, 0.5 or 3 mg/kg leptin daily for 2 weeks. After treatment, serum leptin levels, serum testosterone levels, sperm parameters and testicular cell apoptosis were evaluated. Blood testis barrier integrity and the expression of tight junction-associated proteins in testes were also assessed. We further verified the direct effects of leptin on tight junction-associated proteins in Sertoli cells and the possible leptin signaling pathways involved in this process.

**Results:**

After treatment, there were no significant differences in body weights, reproductive organ weights, serum leptin levels and serum testosterone levels between leptin-treated mice and control mice. Administration of 3 mg/kg leptin reduced sperm concentration, motility and progressive motility while increasing the percentage of abnormal sperm and testicular cell apoptosis. Mice treated with 3 mg/kg leptin also had impaired blood testis barrier integrity, which was related to decreased tight junction-associated proteins in testes. Leptin directly reduced tight junction-associated proteins in Sertoli cells, JAK2/STAT, PI3K and ERK pathways were suggested to be involved in this process.

**Conclusions:**

Exogenous leptin negatively affects sperm parameters and impairs blood testis barrier integrity in mice. Leptin reduced tight junction-associated proteins in Sertoli cells, indicating that leptin has a direct role in impairing blood testis barrier integrity. Given the function of blood testis barrier in maintaining normal spermatogenesis, leptin-induced blood testis barrier impairment may be one of the mechanisms contributing to male subfertility and infertility.

**Electronic supplementary material:**

The online version of this article (10.1186/s12958-018-0368-4) contains supplementary material, which is available to authorized users.

## Background

Leptin is a 16 kDa peptide product of the *ob* gene and is secreted by the adipose tissue [1]. It binds to leptin receptors (OB-R) to mediate several signaling pathways, including Janus kinase 2/signal transducers and activators of transcription (JAK2/STAT), extracellular signal-regulated kinase (ERK) and phosphoinositide 3-kinase (PI3K) [2]. Leptin has a role in energy homeostasis, glucose and lipid metabolism, and immune and neuroendocrine function that has been shown in both humans and rodents [3]. Leptin is able to restore fertility in *ob*/*ob* mice which are leptin deficient, obese and infertile, indicating that leptin serves as a permissive signal to the reproductive system [4, 5]. Certainly, there is increasing evidence that leptin participates in many events in reproduction [1].

Serum leptin levels are higher in most obese people and in rodents that have ingested the high-fat diet for a long-term [6, 7]. Obese men also have higher seminal leptin levels which are associated with increased serum leptin levels [8]. Body mass index (BMI) has positive correlations with serum leptin levels; both BMI and serum leptin levels correlate positively with abnormal sperm morphology, and correlate negatively with sperm concentration and motility [9, 10]. This supports the concept that serum leptin mediates a link between obesity and male infertility [10]. Moreover, serum leptin levels are also increased in azoospermic men compared with normozoospermic fertile men [11]. This elevation is not gonadotropin dependent, indicating that leptin has a direct effect on testis function, especially on spermatogenesis [11]. Animal studies have provided evidence that leptin negatively affects male reproduction. Hyperleptinemia has been found to inhibit testicular steroidogenesis and halt testicular maturation in rodents [12, 13].

Administration of exogenous leptin decreased sperm count and increased the percentage of abnormal sperm in nonobese rodents, suggesting that leptin plays a role in the negative correlations between BMI and sperm quantity and quality [14]. In nonobese rodents, some studies have also shown that exogenous leptin can increase the percentage of abnormal sperm and the DNA fragmentation level while decreasing sperm count and motility, histone to protamine transition during spermatogenesis, and the ability to generate offspring [15–19]. Leptin may exhibit a direct effect on testicular tissues or spermatozoa leading to abnormal sperm parameters [14]. It may also induce reactive oxygen species (ROS) production and hormone profile modulation to affect male fertility [15]. However, additional research is needed to further clarify the mechanisms of leptin’s negative effects on male reproductive function.

Leptin secreted by visceral adipose tissue has been reported to increase the permeability of the intestinal epithelial barrier by reducing the expression of tight junction (TJ)-associated proteins such as zona occludens-1 (ZO-1), zona occludens-3 (ZO-3), claudin 5 and occludin [20–22]. In addition to be the primary structure of the intestinal epithelial barrier, TJ is also a vital structure of the blood testis barrier (BTB). The BTB is comprised of coexisting TJ, basal ectoplasmic specialization, gap junction and desmosome [23]. TJ in the BTB has two main functions, restricting the passage of molecules and dividing the seminiferous epithelium into basal and apical compartments [24]. In mice, the contribution of occludin and claudins to BTB integrity are determined by deletion of occludin gene or genes for transcription factors that are upstream regulators of claudins [25]. The BTB creates a specialized microenvironment that is necessary for germ cells development and movement [24]. Damage to the BTB can cause germ cell loss, reduced sperm count, male infertility or subfertility [23, 26–28]. As leptin impairs TJ integrity in the intestinal epithelium, and because the impact of leptin on BTB integrity has not been addressed in previous studies, we supposed that leptin might affect male reproduction by impairing BTB integrity.

In this study, we administered different doses of leptin or same volume of saline as a control to adult male mice for 2 weeks. We examined the effects of exogenous leptin on serum leptin levels, serum testosterone levels, sperm parameters and testicular cell apoptosis, as well as BTB integrity and TJ-associated proteins. To evaluate whether leptin had a direct effect on TJ-associated proteins, we treated TM4 cells (a mouse Sertoli cell line) with leptin and further investigated the possible leptin-mediated signaling pathways involved in this process.

## Methods

### Animals and treatments

Seven-week-old male C57BL/6 mice were purchased from Hubei Research Center of Laboratory Animals. Mice were kept under a 12 h light and 12 h darkness cycle at 24 °C and allowed to adapt for 1 week before the experiments. At the age of 8 weeks, mice received daily intraperitoneal injections with 0.1, 0.5 or 3 mg/kg leptin (recombinant mouse leptin, Prospec, Israel) dissolved in saline or same volume of saline as a control for 2 weeks. The weights of mice and food in each cage were measured every 2 days.

Mice were sacrificed by exsanguination under anesthesia the day after treatment ended. Reproductive organs including testes and epididymides were weighed immediately and used for further experiments. Blood samples were collected and stored at room temperature for 1 h to clot, before centrifuging at 3000 rpm for 15 min to obtain serum for ELISA.

All animal experiments were approved by the Tongji Medical College Committee on the Use and Care of Animals and were conducted according to the Committee’s guidelines.

### Cell culture and treatment

TM4 cells were obtained from ATCC and stored in Family Planning Research Institute of Tongji Medical College. TM4 cells were cultured in DMEM/F12 supplemented with 2.5% fetal bovine serum and 5% equine serum at 37 °C and 5% CO_2_.

To detect the direct effects of leptin on TJ-associated proteins in TM4 cells, cells were seeded at a density of 1 × 10^5^/ml in 6-well dishes, cultured with low-serum medium containing 0, 10 or 100 nM leptin for 48 h and then harvested for further experiments. Inhibitors of leptin signaling mediators were employed to determine the possible leptin-mediated signaling pathways in vitro. Cells were pretreated with low-serum medium containing 10 μM AG490, LY294002 or U0126 (the inhibitors of JAK2, PI3K and ERK, respectively) (MCE, USA) dissolved in dimethyl sulfoxide (DMSO) for 4 h (the final concentration of DMSO was 0.1%). The inhibitors were removed, and cells were washed with pre-warmed phosphate buffered saline (PBS). Low-serum medium containing 100 nM leptin was then added to cells. After cultivation for 48 h, cells were harvested for further experiments. The concentration of AG490, LY294002 and U0126 was chosen according to earlier studies [29–31].

### Measurement of serum leptin and testosterone levels

Mouse serum leptin and testosterone levels were measured using commercial ELISA kits from Boster Biological Technology (Wuhan, China) and Cusabio (Wuhan, China), respectively. The measurements were processed according to the manufacturer’s protocols.

### Assessment of sperm parameters

Cauda epididymides from each mouse were dissected in 1 ml pre-warmed Ham’ s F10 buffer (Sigma-Aldrich, USA) and incubated at 37 °C for 15 min to allow spermatozoa to swim out. Sperm concentration, motility and progressive motility were determined according to the 5th WHO laboratory manual guidelines [32]. For the detection of sperm with abnormal morphology, sperm suspensions were smeared on glass slides, allowed to dry, and then fixed and stained using a Diff-Quick kit (Phygene, Fuzhou, China) according to the manufacturer’s protocol. The slides were viewed under a light microscope. At least 200 spermatozoa from each sample were assessed. Abnormalities in sperm morphology including head, tail and head-neck connection abnormalities were determined according to Ward et al. [33].

### Apoptosis of testicular cells

TUNEL assay was conducted to detect testicular cell apoptosis. Sections (5 μm) from frozen testes were deproteinized using proteinase K for 25 min at 37 °C. After blocking with 0.1% Triton X-100 for 20 min at room temperature, sections were incubated with TUNEL working solutions (Roche, Germany) in the dark at 37 °C for 1 h and then stained with DAPI and mounted in glycerin. Sections were observed using a fluorescent microscope, and TUNEL positive nuclei which indicated apoptosis were counted in at least 40 seminiferous tubules from three non-consecutive testis sections from each mouse.

### Biotin tracer experiment

The biotin tracer experiment was used to determine BTB integrity according to the method of Meng et al. [34], with a minor modification. EZ-Link Sulfo-NHS-LC-Biotin (Thermo Scientific, USA) was freshly diluted in PBS containing 1 mM CaCl_2_ at a final concentration of 10 mg/ml. Mice were anesthetized, and their testes were exposed. A 30G needle was used to gently inject 30 μl of biotin solution into the testes. After 30 min, mice were euthanized, and their testes were immediately removed and frozen. Sections (5 μm) from frozen testes were blocked with 5% albumin from bovine serum in PBS containing 0.1% Triton X-100 for 1 h, and then incubated with streptavidin conjugate-Alexa Fluor 568 (1:3000, Invitrogen, USA) for 30 min at room temperature. Finally, sections were stained with DAPI, mounted in glycerin and observed using a fluorescent microscope. At least 30 seminiferous tubules from three non-consecutive testis sections from each mouse were examined.

### Western blot

Western blot was used to detect the expression of TJ-associated proteins both in testes and in TM4 cells, and was performed according to the standard procedure. Antibodies against claudin 5 (Invitrogen, USA), occludin (Proteintech, USA), ZO-1 (Proteintech, USA) and β-Actin (Proteintech, USA) were used with the details given in Additional file 1: Table S1. Immunopositive bands were detected using the enhanced chemiluminescence (ECL) (Beyotime, Beijing, China). β-Actin served as the loading control. The densitometric analysis was performed using ImageJ software.

### Immunofluorescence

Immunofluorescence was used to detect the expression and localization of TJ-associated proteins in sections (5 μm) from frozen testes, and was conducted according to the standard procedure. Antibodies against claudin 5 (Invitrogen, USA), occludin (Proteintech, USA) and ZO-1 (Proteintech, USA) were used with the details given in Additional file 1: Table S1. After incubated with Alexa Fluor 488-conjugated secondary antibodies (1:200, Proteintech, USA), sections were stained with DAPI, mounted in glycerin and observed by a fluorescent microscope.

### RNA isolation and PCR

Total RNA in TM4 cells and testes was extracted using TRIzol reagent (Invitrogen, USA), and was then reversed-transcribed into cDNA using a PrimeScript RT reagent kit (TAKARA, Japan). To detect leptin receptor mRNA in TM4 cells, synthesized cell cDNA was subjected to PCR using Premix Taq (TAKARA, Japan). PCR products were run in 1.5% agarose gel electrophoresis (120 V, 30 min) and visualized using an imaging system (Bio-Rad, USA). To determine the expression of steroidogenic genes (*Sf-1*, *Star* and *Cyp11a1*) and androgen receptor in testes, synthesized testicular cDNA was subjected to real time quantitative PCR using SYBR ® Premix Ex Taq II (TAKARA, Japan). The primer sequences were listed in Additional file 2: Table S2. Primers for leptin receptor and *Sf-1* have been reported by El-Hefnawy et al. [35] and Woods et al. [36], respectively.

### Statistical analysis

All data were analyzed using SPSS (ver.21) software. Differences between groups were determined using Kruskal-Wallis test, one-way ANOVA followed by Dunnett test, chi-square test or Student’s t test. Data were expressed as mean ± SD, and differences were considered significant when *p* < 0.05. Graphs were made using Graphpad Prism 7.

## Results

### Leptin administration did not significantly alter serum leptin and testosterone levels in mice

**Fig. 1:**
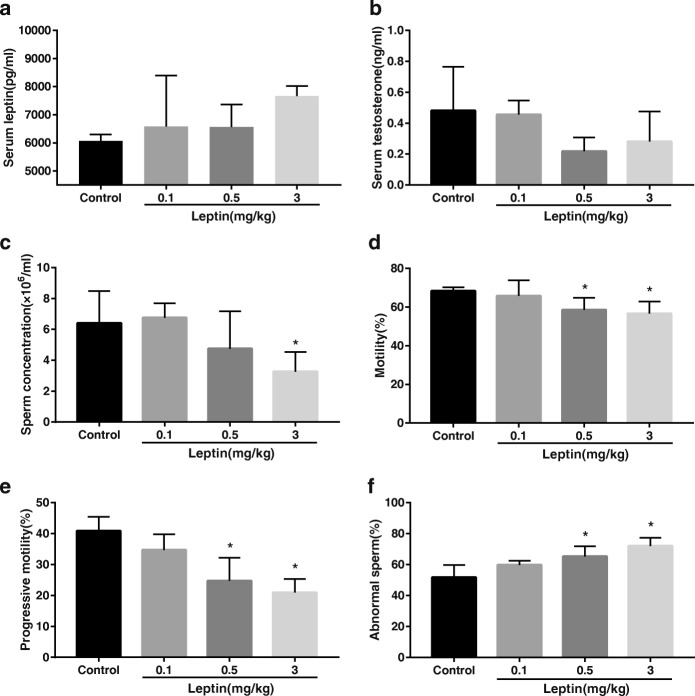
Serum leptin, serum testosterone and sperm parameters in mice. **a** serum leptin. **b** serum testosterone. Data are expressed as mean ± SD, *n* = 5. **c** sperm concentration. **d** sperm motility. **e** sperm progressive motility. **f** percentage of abnormal sperm (sperm with abnormal morphology). Data are expressed as mean ± SD, *n* = 8. * versus control, *p* < 0.05

After 2 weeks of leptin administration, serum leptin and testosterone levels in the leptin-treated groups showed no significant differences compared with the control group (Fig. 1a and b). However, the expression of testicular steroidogenic genes such as steroidogenic factor 1 (*Sf-1*), steroidogenic acute regulatory protein (*Star*) and cytochrome P450 family 11 subfamily A member 1 (*Cyp11a1*) were significantly downregulated in mice treated with a relatively high dose of leptin (3 mg/kg) compared with control mice (*p < 0.05*) (Additional file 3: Figure S1 E).

Throughout the experiment, there were no significant differences in body weights, food intake or reproductive organ weights between the leptin-treated groups and the control group (Additional file 3: Figure S1 A–D).

### Leptin administration altered sperm parameters and increased testicular cell apoptosis

Sperm concentration in the 3 mg/kg leptin-treated group decreased by 50.90% compared with the control group (3.26 ± 1.27 vs. 6.41 ± 2.06 × 10^6^/ml, *p* < 0.05) (Fig. 1c). Sperm motility was 68.38 ± 1.87% in the control group but was lower at 58.57 ± 6.24% in the 0.5 mg/kg leptin-treated group and 56.60 ± 6.32% in the 3 mg/kg leptin-treated group (both *p* < 0.05) (Fig. 1d). Sperm progressive motility in the 0.5 (24.71 ± 7.49%) and 3 mg/kg (20.93 ± 4.43%) leptin-treated groups also decreased significantly compared with the control group (40.84 ± 4.55%) (both *p* < 0.05) (Fig. 1e). The 0.5 and 3 mg/kg leptin-treated groups both had higher proportions of spermatozoa with abnormal morphology, which were 1.25-fold (65.31 ± 6.51%) and 1.38-fold (72.05 ± 5.30%) compared with the control group (51.80 ± 8.01%), respectively (both *p* < 0.05) (Fig. 1f). Administration of 0.1 mg/kg leptin did not alter sperm parameters significantly.

**Fig. 2:**
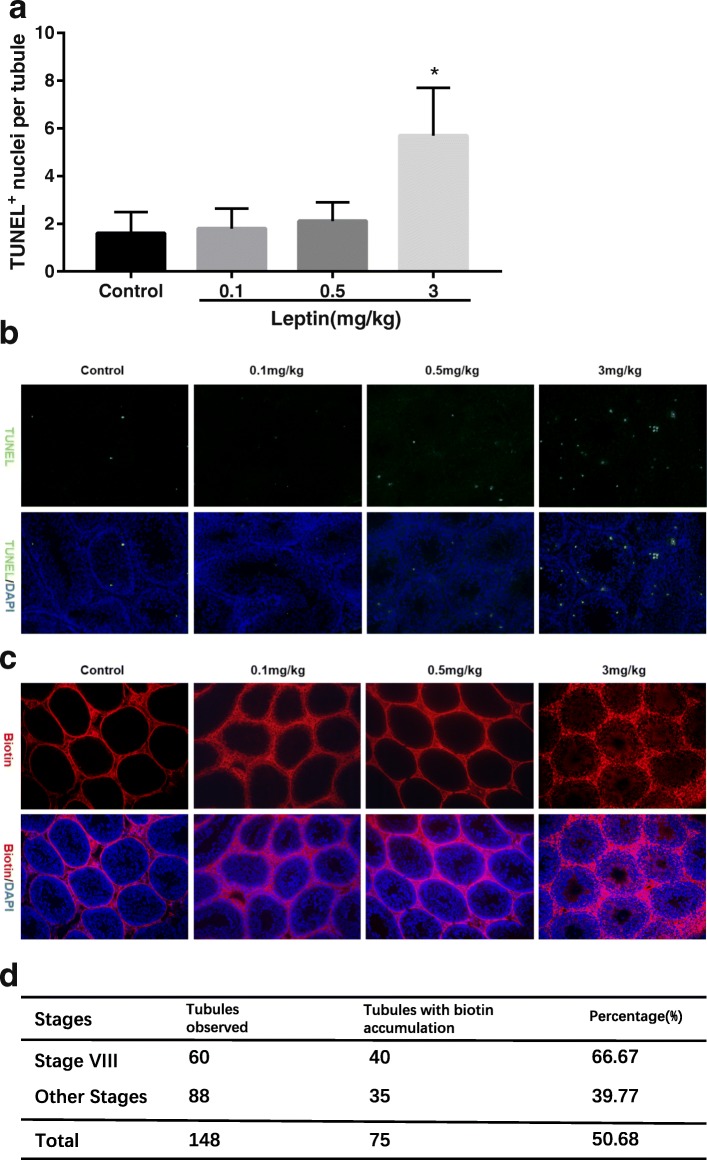
Evaluation of testicular cell apoptosis and BTB integrity. **a** the number of TUNEL positive nuclei per seminiferous tubule. Data are expressed as mean ± SD, *n* = 3. * versus control, *p* < 0.05. **b** TUNEL positive nuclei (green) which indicated apoptosis were mainly localized in germ cells in seminiferous tubules. Cell nuclei were stained with DAPI (blue). **c** biotin (red) only passed through the BTB and accumulated in the adluminal compartments of seminiferous tubules in 3 mg/kg leptin-treated mice. Cell nuclei were stained with DAPI (blue). **d** proportion of seminiferous tubules that have the biotin in the adluminal compartments in all observed seminiferous tubules at stage VIII and other stages. *n* = 4. χ^2^ = 10.323, *p* < 0.05

TUNEL was conducted to detect whether leptin treatment induced testicular cell apoptosis. The number of TUNEL positive nuclei (indicating apoptotic cells) per seminiferous tubule increased significantly in the 3 mg/kg leptin-treated group compared with the control group (*p < 0.05*), and it seemed that apoptosis mainly occurred in germ cells in seminiferous tubules. The number of apoptotic cells per seminiferous tubule in the control, 0.1 and 0.5 mg/kg leptin-treated groups were similar (Fig. 2a and b).

### Leptin administration impaired BTB integrity

We used a biotin tracer to assess if BTB integrity was affected in leptin-treated mice. Biotin passed through the BTB and accumulated visibly in the adluminal compartments of most seminiferous tubules in 3 mg/kg leptin-treated mice, indicating impaired BTB integrity in these mice. In contrast, biotin was restricted to the interstitial and seminiferous tubule-basal compartments in control mice, as well as in 0.1 and 0.5 mg/kg leptin-treated mice (Fig. 2c).

Interestingly, we also observed that seminiferous tubules at stage VIII, of which the BTB undergoes restructuring to allow the transit of preleptotene spermatocytes, more often have the biotin in the adluminal compartments compared with seminiferous tubules at other stages. The proportion of seminiferous tubules that have the biotin in the adluminal compartments in all observed seminiferous tubules at stage VIII and other stages were 66.67% and 39.77%, respectively (χ^2^ = 10.323, *p* < 0.05) (Fig. 2d).

### Leptin administration reduced TJ-associated proteins in testes

**Fig. 3:**
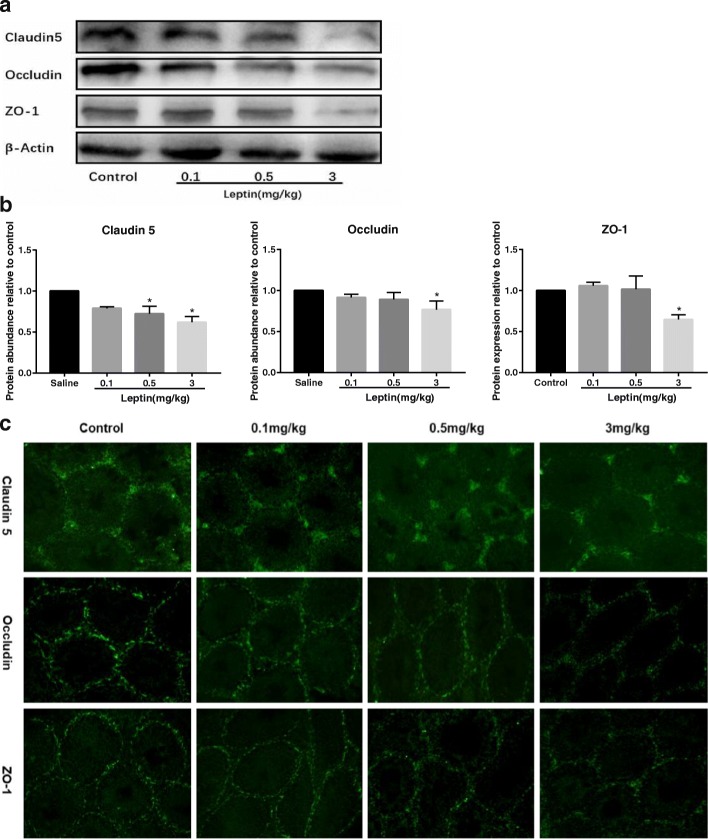
Expression and localization of TJ-associated proteins in testes. **a** western blot analysis of claudin 5, occludin and ZO-1 in testes. **b** densitometric analysis for immunopositive bands of claudin 5, occludin and ZO-1 in testes. Data are expressed as mean ± SD, *n* = 6. * versus control, *p* < 0.05. **c** immunofluorescence showed the expression and localization of claudin 5, occludin and ZO-1 (green) in testes

We determined whether impaired BTB integrity was related to decreased expression of TJ-associated proteins, as TJ restricts the passage of molecules at this barrier. Western blot results demonstrated that the expression of testicular claudin 5, occludin and ZO-1 in 3 mg/kg leptin-treated mice, which had impaired BTB integrity, decreased significantly compared with control mice (*p* < 0.05) (Fig. 3a and b). In control mice, immunofluorescence showed that claudin 5, occludin and ZO-1 were located at the basal compartments of seminiferous tubules, consistent with their expression locations at BTB area, and claudin 5 was simultaneously expressed in germ cells and in vascular endothelium. However, the immunofluorescent stains of these proteins at BTB area became thin and irregular in 3 mg/kg leptin-treated mice (Fig. 3c). In addition, androgen receptor (AR) is reported to be an upstream factor affecting BTB integrity. We found that testicular AR expression in 3 mg/kg leptin-treated mice decreased significantly compared with control mice (*p* < 0.05) (Additional file 3: Figure S1 F).

### Leptin directly reduced TJ-associated proteins in TM4 cells

**Fig. 4:**
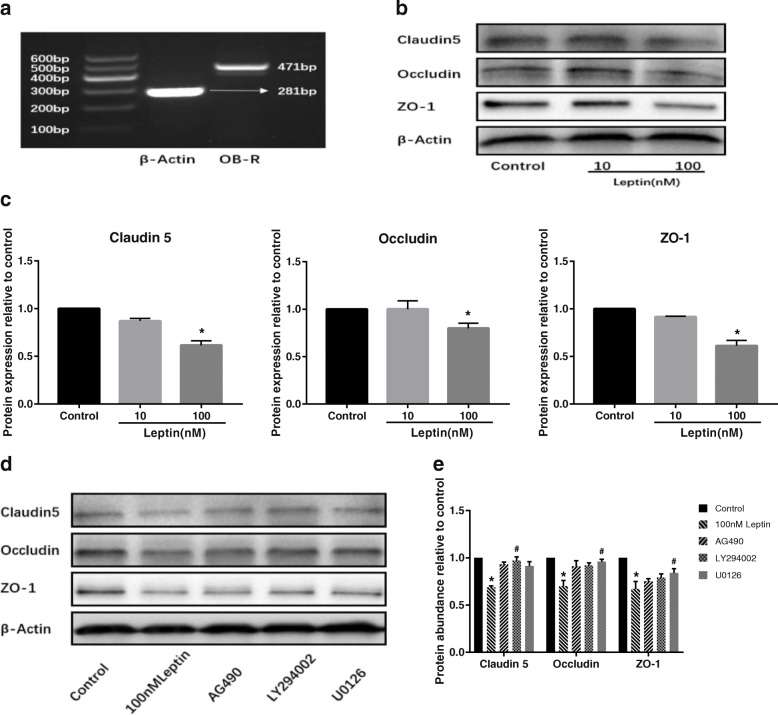
Leptin directly reduced the expression of TJ-associated proteins in vitro, and inhibitors of leptin signaling mediators abolished leptin’s effect to different degrees. **a** detection of OB-R in TM4 cells, the bands of 471 bp and 281 bp corresponded to OB-R and β-Actin, respectively. **b** western blot analysis of claudin 5, occludin and ZO-1 in TM4 cells after treated with 0 (control), 10 or 100 nM leptin for 48 h. **c** densitometric analysis for immunopositive bands of claudin 5, occludin and ZO-1 in TM4 cells. **d** western blot analysis of claudin 5, occludin and ZO-1 in TM4 cells, cells were treated with 100 nM leptin or pre-treated with different inhibitors following a 100 nM leptin treatment. **e** densitometric analysis for immunopositive bands of claudin 5, occludin and ZO-1 in inhibitor assay. Data are expressed as mean ± SD, n = 5. * versus control, ^#^ versus 100 nM leptin, *p* < 0.05

To identify whether leptin could directly reduce TJ-associated proteins in Sertoli cells in vitro, we treated TM4 cells, an OB-R expressing mouse Sertoli cell line (Fig. 4a), with 0 (control), 10 or 100 nM leptin for 48 h. We found that the expression of claudin 5, occludin and ZO-1 decreased significantly in cells treated with 100 nM leptin compared with control cells (*p* < 0.05). The presence of 10 nM leptin showed no significant influence on the expression of TJ-associated proteins in TM4 cells (Fig. 4b and c).

### Leptin’s effect on TJ-associated proteins in TM4 cells was attenuated by leptin signaling pathway inhibitors

We further investigated the requirement of leptin-mediated signaling pathways for reducing TJ-associated proteins in TM4 cells. Various inhibitors of leptin signaling mediators were used in this study: AG490, LY294002 and U0126 (the inhibitors of JAK2, PI3K and ERK, respectively). The decreased expression of claudin 5, occludin and ZO-1 in TM4 cells induced by 100 nM leptin was reversed in various degrees when cells were pretreated with inhibitors. Leptin’s effect on claudin 5 was significantly reduced by LY294002, and U0126 was the most effective inhibitor to abolish leptin’s effect on occludin and ZO-1 (both *p* < 0.05) (Fig. 4d and e). The results indicated that JAK2/STAT, PI3K and ERK pathways were involved in leptin-induced decline in TJ-associated proteins in TM4 cells.

## Discussion

Leptin is a well-known protein secreted by adipose tissue that maintains normal reproductive function. This is proven by administering leptin to *ob*/*ob* mice to restore fertility [4, 5]. However, leptin seems to have adverse impacts on male fertility when serum leptin levels are higher than normal and in nonobese rodents given exogenous leptin. The present study highlighted the effects of exogenous leptin on sperm parameters and the role of leptin in damaging BTB integrity, which could be a mechanism for leptin-related male subfertility and infertility.

It is evident that leptin treatment restores reproductive function in *ob*/*ob* mice [4, 5, 37]. However, the effects of leptin treatment on normal rodents are negative [14–19]. In our study, sperm concentration, motility and progressive motility decreased whereas the percentage of abnormal sperm and the number of apoptotic testicular cells increased in 3 mg/kg leptin-treated mice. Using a biotin tracer, we showed that these mice also had impaired BTB integrity. Haron et al. suggested that decreased sperm count and increased abnormal spermatozoa in leptin-treated rodents were likely due to a direct effect of leptin on spermatozoa or testicular tissues [14]; Abbasihormozi et al. proposed that exogenous leptin suppressed male fertility by sperm ROS production or hormone modulation [15]. Here, we showed that altered sperm parameters in normal mice exposed to exogenous leptin had a relationship with impaired BTB integrity. The BTB acts as a physical and immunological barrier to protect spermatogenic cells from toxicants, and from being recognized and attacked by the immune system [23]. Exposure to some environmental toxicants can induce injury to the BTB and elicit subsequent damage as germ cell loss, reduced sperm count, male infertility or subfertility [23, 26–28]. Impaired BTB might alter the microenvironment for spermatogenesis in 3 mg/kg leptin-treated mice leading to germ cell apoptosis and compromised sperm quantity and quality. The biotin tracer assay also showed that biotin was more often observed in the adluminal compartments of seminiferous tubules at stage VIII. Although the BTB disassembles and reconstructs to facilitate the transit of preleptotene spermatocytes into the apical compartments at this stage, it still holds intact function due to its distinctive structure under normal condition [23]. The results of biotin tracer assay suggested that leptin might interfere with the reconstruction process of the BTB at stage VIII, and thus the BTB was more often impaired at this stage.

The BTB is formed largely by TJ between Sertoli cells, which serves as a barrier and boundary in the seminiferous epithelium. We hypothesized that impaired BTB integrity was associated with decreased expression of TJ-associated proteins. As expected, in 3 mg/kg leptin-treated mice, which had impaired BTB integrity, the expression of claudin 5, occludin and ZO-1 decreased significantly and the immunofluorescent stains of these proteins became thin and irregular at BTB area. Further studies are needed to fully understand the mechanisms underlying leptin’s disruption to the BTB as other junctions in the BTB have not yet been evaluated. Fan et al. determined AR expression using testicular proteins in diet-induced obese mice and suggested AR as an upstream factor affecting BTB integrity [38]. The decreased testicular AR gene expression in 3 mg/kg leptin-treated mice suggested that it could contribute to inducing BTB impairment. On the other hand, we could not rule out the possibility that leptin directly reduced TJ-associated proteins in Sertoli cells. Leptin carries out its biological effects through OB-R which is expressed in rat Sertoli cells and human Sertoli cells [39, 40]. Since leptin can modulate the nutritional support for spermatogenesis by altering the metabolic behavior of human Sertoli cells [40], we treated TM4 cells with low (10 nM) and high concentration (100 nM) of leptin to test the direct effect of leptin on TJ-associated proteins. We first confirmed the presence of OB-R, which allows leptin to interact with TM4 cells. We then found that 100 nM leptin reduced the expression of claudin 5, occludin and ZO-1 in TM4 cells. Taken together, our in vitro experiments confirmed that leptin alone directly reduced TJ-associated proteins, which could contribute to BTB impairment in vivo.

Leptin uses JAK2/STAT3 as its principle signaling pathway [41], and it also activates ERK and PI3K pathways [2]. Inhibition of JAK2, ERK and PI3K reversed leptin-induced decline in TJ-associated proteins in TM4 cells to different extents (Fig. 4e). However, AG490, an inhibitor of JAK2, was not the most effective inhibitor to rescue the decrease of TJ-associated proteins in TM4 cells. Activation of JAK2/STAT3, along with the activation of PI3K and ERK, is involved in leptin-induced TJ dysfunction in intestinal cells [20]. When SUMO-2/3 specific protease (SENP3) is knocked down, it compromises the activation of STAT3, resulting in TJ dysfunction in Sertoli cells [42]. In addition, a high level of leptin has also been found to upregulate the expression of suppressor of cytokine signaling 3 (SOCS3), which can inhibit STAT3 phosphorylation [12]. The role of the JAK2/STAT3 pathway in leptin-induced decline in TJ-associated proteins required further investigation. In this study, JAK2/STAT, PI3K and ERK pathways were suggested to be involved in leptin-induced decline in TJ-associated proteins.

Leptin treatment causes body weight loss and increases reproductive organ weights in *ob*/*ob* mice [4]. However, leptin treatment hardly alters body weights or reproductive organ weights in normal rodents [14, 16–19, 43], also shown in this study, indicating that the effect on sperm parameters after leptin administration is unlikely due to leptin resistance [14]. Previous studies have reported that leptin treatment has no influence on serum leptin levels in normal rodents [14, 18]. Although circulating leptin levels at 1 h after administration of 3 mg/kg leptin show a 170-fold increase in fasted mice and a 13-fold increase in fed mice, the half-life of mouse leptin is found to be 40.2 min [44]. This could explain why serum leptin levels in our leptin-treated mice were not significantly different compared with control mice.

The relationship between leptin treatment and testosterone has been investigated in many studies. In *ob*/*ob* mice, leptin treatment increases intratesticular testosterone via improved Leydig cell function [37]. However, serum testosterone is negatively correlated with serum leptin in humans and rodents [45–47]. In vitro experiments also show that leptin can directly reduce testosterone secretion and the expression of steroidogenic genes [12, 39, 48]. In normal rats, leptin treatment does not change serum testosterone significantly [14, 18], although parenchymal testosterone decreases by about 49% compared with control rats [18]. Our study showed that 3 mg/kg leptin treatment repressed testicular steroidogenesis genes expression in vivo but did not produce a significant decrease in serum testosterone levels. The conflicting findings observed in various studies are most likely due to different experimental objectives and variable study designs such as the different doses of leptin used and experiment durations. In our study, altered sperm parameters and impaired BTB integrity observed in leptin-treated mice were unlikely to be related to the alterations in serum testosterone levels since the decreases were not statistically different. Instead, it seemed to be leptin that exerted critical and direct effects on male reproductive tissues.

## Conclusions

The present study shows that exogenous leptin exhibits significant adverse effects on sperm parameters, induces testicular cell apoptosis, and possibly suppresses testicular steroidogenesis. Exogenous leptin impairs BTB integrity in vivo, which is likely to be a result of decreased TJ-associated proteins. We have further verified that leptin can directly reduce TJ-associated proteins in Sertoli cells in vitro, and identified that JAK2/STAT, PI3K and ERK pathways may be involved in this process. Given the pivotal role of BTB integrity in maintaining an appropriate microenvironment for normal spermatogenesis, BTB impairment may cause male subfertility and infertility. We have proposed a mechanism for leptin’s adverse effects on male reproductive function, which will help to have a deeper insight into subfertility and infertility in the context of obesity and azoospermia.

## Additional files

Additional file 1: **Table 1.** Antibodies used for western blot and immunofluorescence in this study. (XLSX 8 kb)

Additional file 2: **Table 2.** Primers used for PCR in this study. (XLSX 9 kb)

Additional file 3: **Figure S1.** Body weights, food intake, reproductive organ weights, the expression of testicular steroidogenic genes and the expression of testicular AR and OB-R in mice. A, body weights. B, food intake. C, testis weights. D, epididymis weights. Data are expressed as mean ± SD, *n* = 8. E, testicular steroidogenic genes expression. F, testicular AR and OB-R expression. Data are expressed as mean ± SD, *n* = 5. * versus control, *p* < 0.05. (TIF 1202 kb)

## Abbreviations

AR: Androgen receptor
BMI: Body mass index
BTB: Blood testis barrier
*Cyp11a1*: Cytochrome P450 family 11 subfamily A member 1
ERK: Extracellular signal-regulated kinase
JAK2: Janus kinase 2
OB-R: Leptin receptor
PI3K: Phosphoinositide 3-kinase
ROS: Reactive oxygen species
*Sf-1*: Steroidogenic factor 1
SOCS3: Suppressor of cytokine signaling 3
*Star*: Steroidogenic acute regulatory protein
STAT3: Signal transducer and activator of transcription 3
TJ: Tight junction
TUNEL: TdT-mediated dUTP Nick End Labeling
ZO-1: Zona occludens-1
ZO-3: Zona occludens-3

## References

[1] González RR, Simón C, Caballero-Campo P, Norman R, Chardonnens D, Devoto L, Bischof P (2000). Leptin and reproduction. Hum Reprod Update.

[2] Zhou Y, Rui L (2013). Leptin signaling and leptin resistance. Front Med.

[3] Ahima RS (2006). Adipose tissue as an endocrine organ. Obesity.

[4] Barash IA, Cheung CC, Weigle DS, Ren H, Kabigting EB, Kuijper JL, Clifton DK, Steiner RA (1996). Leptin is a metabolic signal to the reproductive system. Endocrinology.

[5] Mounzih K, Lu R, Chehab FF (1997). Leptin treatment rescues the sterility of genetically obese Ob/Ob males. Endocrinology.

[6] Considine RV, Sinha MK, Heiman ML, Kriauciunas A, Stephens TW, Nyce MR (1996). Serum immunoreactive-leptin concentrations in normal-weight and obese humans. N Engl J Med.

[7] Handjieva-Darlenska T, Boyadjieva N (2009). The effect of high-fat diet on plasma ghrelin and leptin levels in rats. J Physiol Biochem.

[8] Leisegang K, Bouic PJ, Menkveld R, Henkel RR (2014). Obesity is associated with increased seminal insulin and leptin alongside reduced fertility parameters in a controlled male cohort. Reprod Biol Endocrinol.

[9] Einollahi N, Dashti N, Emamgholipour S, Zarebavani M, Sedighi-Gilani MA, Choobineh H (2016). Evidence for alteration in serum concentrations of leptin in infertile men categorized based on BMI. Clin Lab.

[10] Hofny ER, Ali ME, Abdel-Hafez HZ, Eel-D K, Mohamed EE, Abd El-Azeem HG, Mostafa T (2010). Semen parameters and hormonal profile in obese fertile and infertile males. Fertil Steril.

[11] Steinman N, Gamzu R, Yogev L, Botchan A, Schreiber L, Yavetz H (2001). Serum leptin concentrations are higher in azoospermic than in normozoospermic men. Fertil Steril.

[12] Yuan M, Huang G, Li J, Zhang J, Li F, Li K (2014). Hyperleptinemia directly affects testicular maturation at different sexual stages in mice, and suppressor of cytokine signaling 3 is involved in this process. Reprod Biol Endocrinol.

[13] Giovambattista A, Suescun MO, Nessralla CC, França LR, Spinedi E, Calandra RS (2003). Modulatory effects of leptin on leydig cell function of normal and hyperleptinemia rats. Neuroendocrinology.

[14] Haron MN, D'Souza UJ, Jaafar H, Zakaria R, Singh HJ (2010). Exogenous leptin administration decreases sperm count and increases the fraction of abnormal sperm in adult rats. Fertil Steril.

[15] Abbasihormozi S, Shahverdi A, Kouhkan A, Cheraghi J, Akhlaghi AA, Kheimeh A (2013). Relationship of leptin administration with production of reactive oxygen species, sperm DNA fragmentation, sperm parameters and hormone profile in the adult rat. Arch Gynecol Obstet.

[16] Almabhouh FA, Osman K, Ibrahim SF, Gupalo S, Gnanou J, Ibrahim E, Singh HJ (2016). Melatonin ameliorates the adverse effects of leptin on sperm. Asian J Androl.

[17] Almabhouh FA, Singh HJ (2018). Adverse effects of leptin on histone- to- protamine transition during spermatogenesis are prevented by melatonin in Sprague- Dawley rats. Andrologia.

[18] Fernandez CD, Fernandes GS, Favareto AP, Perobelli JE, Sanabria M, Kempinas WD (2017). Decreased implantation number after in utero artificial insemination can reflect an impairment of fertility in adult male rats after exogenous leptin exposure. Reprod Sci.

[19] Almabhouh FA, Osman K, Siti Fatimah I, Sergey G, Gnanou J, Singh HJ (2015). Effects of leptin on sperm count and morphology in Sprague-Dawley rats and their reversibility following a 6-week recovery period. Andrologia.

[20] Kim CY, Kim KH (2014). Curcumin prevents leptin-induced tight junction dysfunction in intestinal Caco-2 BBe cells. J Nutr Biochem.

[21] Le Dréan G, Haure-Mirande V, Ferrier L, Bonnet C, Hulin P, de Coppet P, Segain JP (2014). Visceral adipose tissue and leptin increase colonic epithelial tight junction permeability via a RhoA-ROCK-dependent pathway. FASEB J.

[22] Le Dréan G, Segain JP (2014). Connecting metabolism to intestinal barrier function: the role of leptin. Tissue Barriers.

[23] Cheng CY, Mruk DD (2012). The blood-testis barrier and its implications for male contraception. Pharmacol Rev.

[24] Mruk DD, Cheng CY (2004). Sertoli-Sertoli and Sertoli-germ cell interactions and their significance in germ cell movement in the seminiferous epithelium during spermatogenesis. Endocr Rev.

[25] Morrow CM, Mruk D, Cheng CY, Hess RA (2010). Claudin and occludin expression and function in the seminiferous epithelium. Philos Trans R Soc Lond Ser B Biol Sci.

[26] Cao XN, Shen LJ, Wu SD, Yan C, Zhou Y, Xiong G (2017). Urban fine particulate matter exposure causes male reproductive injury through destroying blood-testis barrier (BTB) integrity. Toxicol Lett.

[27] Zhang J, Li Z, Qie M, Zheng R, Shetty J, Wang J (2016). Sodium fluoride and sulfur dioxide affected male reproduction by disturbing blood-testis barrier in mice. Food Chem Toxicol.

[28] Qiu L, Zhang X, Zhang X, Zhang Y, Gu J, Chen M (2013). Sertoli cell is a potential target for perfluorooctane sulfonate-induced reproductive dysfunction in male mice. Toxicol Sci.

[29] Ahn JH, Choi YS, Choi JH (2015). Leptin promotes human endometriotic cell migration and invasion by up-regulating MMP-2 through the JAK2/STAT3 signaling pathway. Mol Hum Reprod.

[30] Rossi SP, Windschüttl S, Matzkin ME, Rey-Ares V, Terradas C, Ponzio R (2016). Reactive oxygen species (ROS) production triggered by prostaglandin D2 (PGD2) regulates lactate dehydrogenase (LDH) expression/activity in TM4 Sertoli cells. Mol Cell Endocrinol.

[31] Choi MS, Park HJ, Oh JH, Lee EH, Park SM, Yoon S (2014). Nonylphenol-induced apoptotic cell death in mouse TM4 Sertoli cells via the generation of reactive oxygen species and activation of the ERK signaling pathway. J Appl Toxicol.

[32] World Health Organization (2010). WHO laboratory manual for the examination and processing of human semen.

[33] Ward MA (2005). Intracytoplasmic sperm injection effects in infertile azh mutant mice. Biol Reprod.

[34] Meng J, Holdcraft RW, Shima JE, Griswold MD, Braun RE (2005). Androgens regulate the permeability of the blood–testis barrier. Proc Natl Acad Sci.

[35] El-Hefnawy T, Ioffe S, Dym M (2000). Expression of the leptin receptor during germ cell development in the mouse testis. Endocrinology.

[36] Woods DC, White YA, Niikura Y, Kiatpongsan S, Lee HJ, Tilly JL (2013). Embryonic stem cell–derived granulosa cells participate in ovarian follicle formation in vitro and in vivo. Reprod Sci.

[37] Hoffmann A, Manjowk GM, Wagner IV, Klöting N, Ebert T, Jessnitzer B (2016). Leptin within the subphysiological to physiological range dose-dependently improves male reproductive function in an obesity mouse model. Endocrinology.

[38] Fan Y, Liu Y, Xue K, Gu G, Fan W, Xu Y, Ding Z (2015). Diet-induced obesity in male C57BL/6 mice decreases fertility as a consequence of disrupted blood-testis barrier. PLoS One.

[39] Tena-Sempere M, Manna PR, Zhang FP, Pinilla L, González LC, Diéguez C, Huhtaniemi I, Aguilar E (2001). Molecular mechanisms of leptin action in adult rat testis: potential targets for leptin-induced inhibition of steroidogenesis and pattern of leptin receptor messenger ribonucleic acid expression. J Endocrinol.

[40] Martins AD, Moreira AC, Sá R, Monteiro MP, Sousa M, Carvalho RA (2015). Leptin modulates human Sertoli cell acetate production and glycolytic profile: a novel mechanism of obesity-induced male infertility?. Biochim Biophys Acta.

[41] Peelman F, Zabeau L, Moharana K, Savvides SN, Tavernier J (2014). 20 years of leptin: insights into signaling assemblies of the leptin receptor. J Endocrinol.

[42] Wu D, Huang CJ, Khan FA, Jiao XF, Liu XM, Pandupuspitasari NS, Brohi RD, Huo LJ (2017). SENP3 grants tight junction integrity and cytoskeleton architecture in mouse Sertoli cells. Oncotarget.

[43] Donahoo WT, Stob NR, Ammon S, Levin N, Eckel RH (2011). Leptin increases skeletal muscle lipoprotein lipase and postprandial lipid metabolism in mice. Metabolism.

[44] Burnett LC, Skowronski AA, Rausch R, LeDuc CA, Leibel RL (2017). Determination of the half-life of circulating leptin in the mouse. Int J Obes.

[45] Luukkaa V, Pesonen U, Huhtaniemi I, Lehtonen A, Tilvis R, Tuomilehto J, Koulu M, Huupponen R (1998). Inverse correlation between serum testosterone and leptin in men. J Clin Endocrinol Metab.

[46] Söderberg S, Olsson T, Eliasson M, Johnson O, Brismar K, Carlström K, Ahrén B (2001). A strong association between biologically active testosterone and leptin in non-obese men and women is lost with increasing (central) adiposity. Int J Obes Relat Metab Disord.

[47] Vigueras-Villaseñor RM, Rojas-Castañeda JC, Chávez-Saldaña M, Gutiérrez-Pérez O, García-Cruz ME, Cuevas-Alpuche O (2011). Alterations in the spermatic function generated by obesity in rats. Acta Histochem.

[48] Landry DA, Sormany F, Haché J, Roumaud P, Martin LJ (2017). Steroidogenic genes expressions are repressed by high levels of leptin and the JAK/STAT signaling pathway in MA-10 Leydig cells. Mol Cell Biochem.

