# Supplementary figures and images for: Exogenous leptin affects sperm parameters and impairs blood testis barrier integrity in adult male mice
Source: Reprod Biol Endocrinol. 2018 May 31;16:55. doi: 10.1186/s12958-018-0368-4 (PMC5984414; doi:10.1186/s12958-018-0368-4)

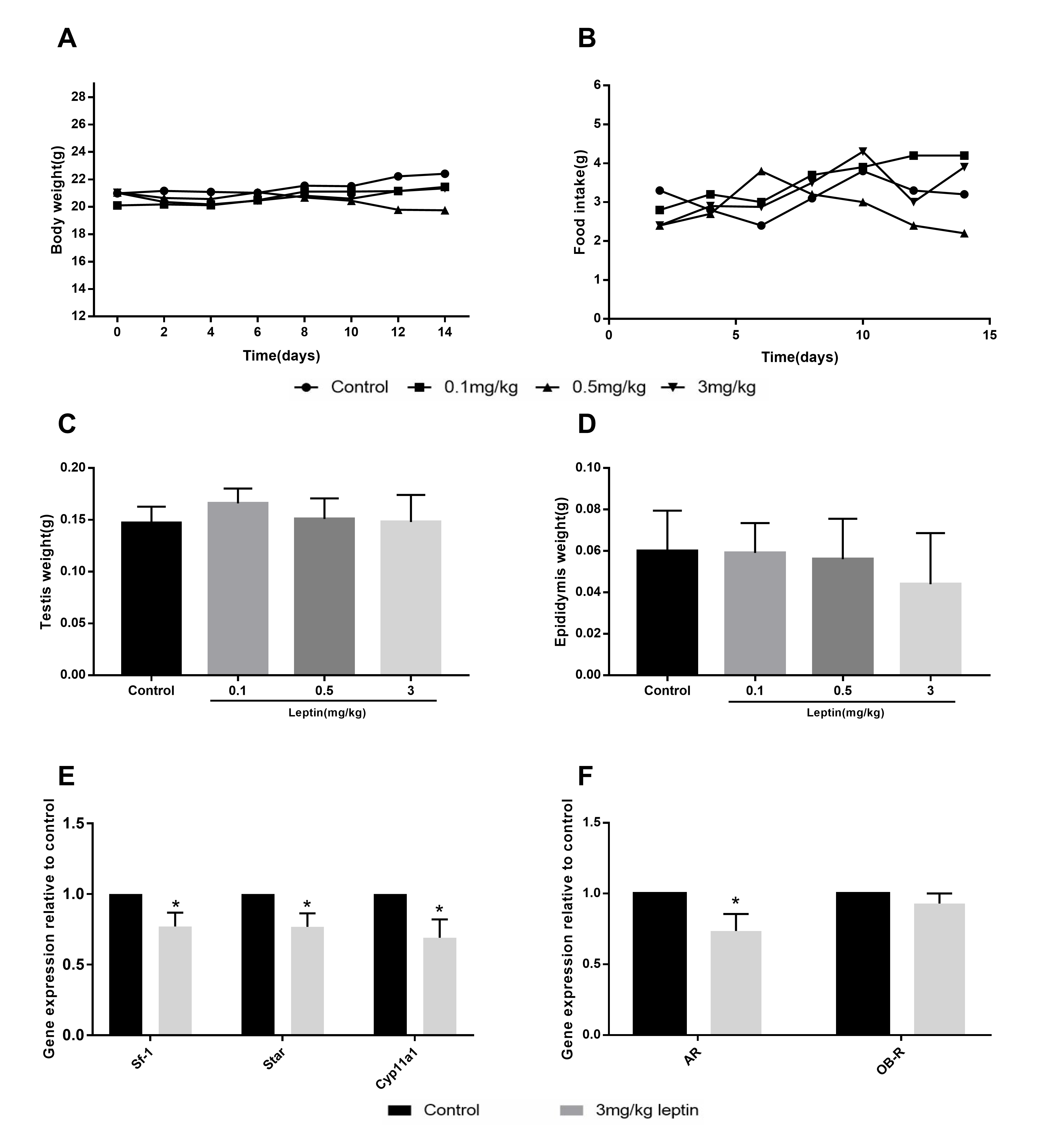

Supplement: Supplementary file 3 — Figure S1. Body weights, food intake, reproductive organ weights, the expression of testicular steroidogenic genes and the expression of testicular AR and OB-R in mice. A, body weights. B, food intake. C, testis weights. D, epididymis weights. Data are expressed as mean ± SD, n = 8. E, testicular steroidogenic genes expression. F, testicular AR and OB-R expression. Data are expressed as mean ± SD, n = 5. * versus control, p < 0.05. (TIF 1202 kb) [file 12958_2018_368_MOESM3_ESM.tif]
